# Nuclear Lamins in Cardiac Development and Disease

**DOI:** 10.3390/cells15090844

**Published:** 2026-05-05

**Authors:** Siqi Li, Rui Li, Chun Liu, Dongzhu Xu, Lu Han

**Affiliations:** 1 Department of Pediatrics, Medical College of Wisconsin, Milwaukee, WI 53226, USA; siqili@mcw.edu (S.L.); s2530552@u.tsukuba.ac.jp (R.L.); 2 Division of Pediatric Cardiology, Herma Heart Institute, Children’s Hospital of Wisconsin, Milwaukee, WI 53226, USA; 3 Cardiovascular Research Center, Medical College of Wisconsin, Milwaukee, WI 53226, USA; chunliu@mcw.edu; 4 Tsukuba Life Science Innovation Program, Graduate School of Comprehensive Human Sciences, University of Tsukuba, 1-1-1 Tennodai, Tsukuba 305-8575, Japan; xu_dongzhu@md.tsukuba.ac.jp; 5 Department of Cardiology, Faculty of Medicine, University of Tsukuba, 1-1-1 Tennodai, Tsukuba 305-8575, Japan; 6 Department of Physiology and Medicine, Medical College of Wisconsin, Milwaukee, WI 53226, USA; 7 Cancer Center, Medical College of Wisconsin, Milwaukee, WI 53226, USA

**Keywords:** lamins, lamin A/C, lamin B1/B2, heart development, cardiomyocyte proliferation and maturation, nuclear mechanics, chromatin organization, cardiomyopathy, lamina-associated domains (LAD), regeneration

## Abstract

Nuclear lamins organize the structural and regulatory architecture of the nucleus, integrating nuclear mechanics, chromatin organization, and genome regulation. During cardiac development, lamin composition undergoes a coordinated transition that parallels the shift from proliferative embryonic cardiomyocytes to mechanically active postnatal cells. Recent findings reveal that B-type lamins support early nuclear plasticity and proliferative capacity, whereas Lamin A/C stabilizes nuclear architecture and transcriptional programs in mature cardiomyocytes. Beyond their structural roles, lamins participate in multiple layers of nuclear regulation, including lamina-associated chromatin organization, nucleo–cytoskeletal mechanotransduction, nucleocytoplasmic transport, and regulation of mitotic progression and cell-cycle exit. Through these interconnected functions, the nuclear lamina coordinates cardiomyocyte proliferation, maturation, and mechanical stress adaptation during heart development. Mutations in lamin genes cause a diverse group of disorders collectively known as laminopathies, many of which prominently affect the cardiovascular system. In this review, we first examine how B-type and A-type lamins are developmentally deployed to regulate cardiomyocyte proliferation and maturation in the heart. We then discuss the mechanistic pathways through which lamins organize nuclear architecture, chromatin dynamics, and nucleo–cytoskeletal signaling to coordinate cardiac cellular function. Finally, we consider how disruption of these lamin-dependent regulatory networks contributes to cardiomyopathy, cardiovascular aging, and the loss of regenerative capacity in the adult mammalian heart.

## 1. Introduction

Lamins are type V intermediate filament proteins that assemble into a filamentous network underlying the inner nuclear membrane [1,2]. Beyond providing structural support to the nucleus, lamins play central roles in genome organization and nuclear regulation, including transcriptional control, DNA replication and repair, and cell-cycle progression [3,4,5,6]. During development, cells face shifting functional demands, ranging from nuclear plasticity and proliferative capacity to long-term mechanical resilience. These transitions raise an important question: how does the nuclear lamina remodel to support the transformation of the heart from a population of proliferating cells into a high-pressure, continuous contracting pump?

In mammals, nuclear lamins are classified into A- and B-type isoforms. The A-type lamins, lamin A and lamin C, are alternatively spliced products of the *LMNA* gene, whereas the B-type lamins, lamin B1 and lamin B2, are encoded by *LMNB1* and *LMNB2*, respectively [7,8,9,10,11,12]. Although lamins were initially defined as structural components of the nuclear envelope, they are now recognized as multifunctional regulators that integrate nuclear mechanics with chromatin organization and gene regulation.

Across metazoans, the composition of the nuclear lamina reflects progressive functional diversification. Invertebrates such as *Caenorhabditis elegans* rely on a single lamin protein that exhibits features of both A- and B-type lamins, consistent with an ancestral multifunctional state [13,14,15]. In contrast, *Drosophila melanogaster* displays an early form of lamin class separation: the B-type lamins (lamin Dm) is constitutively expressed, whereas lamin C, which possesses A-type characteristics, is developmentally regulated and enriched in differentiated tissues [16,17,18]. This diversification becomes further elaborated in vertebrates, including zebrafish, which express distinct A- and B-type lamins [19,20]. Together, these evolutionary patterns suggest that B-type lamins provide a foundational nuclear framework optimized for early developmental plasticity and proliferation, whereas A-type lamins emerged to support the mechanical and regulatory demands of differentiated, long-lived cell states.

The heart is an informative system for studying developmental deployment of lamin classes. Cardiomyocytes undergo a highly staged transition from proliferative embryonic cells to terminally differentiated, mechanically active postnatal cells. This transition involves coordinated cell-cycle withdrawal, polyploidization, structural maturation, and metabolic remodeling [21,22]. Accordingly, lamin composition is dynamically regulated during cardiac development to support both early developmental plasticity and later resistance to sustained mechanical stress. Disruption of this balance underscores the essential role of lamin-mediated nuclear adaptation in the heart.

**Figure 1:**
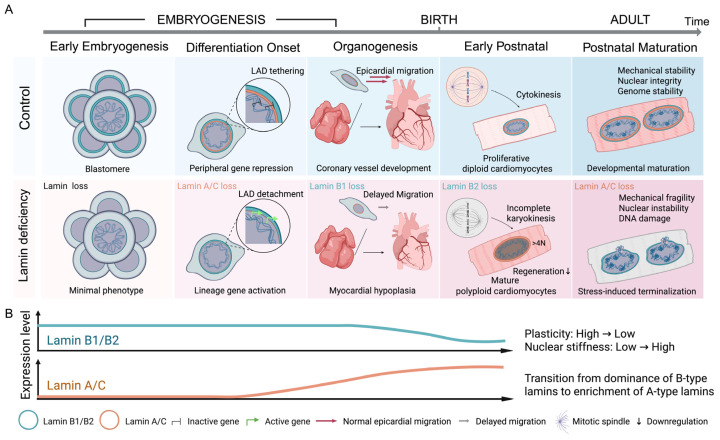
**Developmental deployment and stage-specific functions of nuclear lamins during cardiac development**. (**A**) Nuclear lamins play stage-specific roles during cardiac development. During early embryogenesis, lamin loss produces minimal phenotypic consequences in proliferating blastomeres. At the onset of differentiation, lamina-associated domains (LADs) tether lineage genes to the nuclear periphery to maintain transcriptional repression; lamin deficiency disrupts LAD positioning and promotes premature gene activation. During organogenesis, B-type lamins support nuclear deformation required for epicardial migration and coronary vessel development. lamin B1 loss delays epicardial migration and leads to myocardial hypoplasia. In early postnatal cardiomyocytes, lamin B2 preserves mitotic fidelity, whereas lamin B2 deficiency induces spindle defects, karyokinesis failure, cardiomyocyte polyploidization, and reduced regenerative capacity. During postnatal maturation and adulthood, lamin A/C maintains nuclear integrity under sustained mechanical load; lamin A/C loss results in nuclear instability, DNA damage, and stress-induced cardiomyocyte dysfunction. (**B**) Developmental dynamics of lamin isoforms in the heart. B-type lamins dominate during embryogenesis, whereas lamin A/C progressively increases after birth, reflecting a transition from dominance of B-type lamins to enrichment of A-type lamins. This switch accompanies reduced cellular plasticity and increased nuclear stiffness during cardiomyocyte maturation. Created with BioRender.com.

In this review, we synthesize current knowledge to propose a developmental framework for lamin function in the heart (Figure 1). We discuss how B-type lamins support early nuclear plasticity and proliferative capacity, examine lamin B2 as a key regulator of the cardiomyocyte proliferation-to-maturation transition, and distinguish physiological developmental maturation from stress-induced terminalization associated with lamin A/C dysfunction. We further explore how lamina-associated chromatin organization and lamin A/C-dependent regulatory networks stabilize mature cardiomyocyte identity. Together, this perspective highlights nuclear lamins not merely as structural components but as dynamic regulators of cardiac developmental progression.

Although this review emphasizes cardiac development, the principles discussed, developmental timing, nuclear mechanics, chromatin organization, and mechanical stress adaptation, are broadly conserved across tissues. Evidence from neuron, muscle, and stem cell systems is therefore incorporated where it provides mechanistic insight into general lamin biology.

## 2. Developmental Deployment and Stage-Specific Functions of Lamins in Cardiac Development

Cardiac development in this review is described using several broad developmental stages. Early embryogenesis refers to the initial development following fertilization, where a single-celled zygote divides and differentiates into a multicellular structure. Tissue differentiation denotes the period when stem cells undergo changes in gene expression for lineage specification, becoming specialized cellular identities. Late embryogenesis (organogenesis) corresponds to stages of active cell migration, tissue morphogenesis, and organ assembly. Birth and early postnatal stages mark the transition to extrauterine circulation, accompanied by cardiomyocyte cell-cycle exit and structural maturation. Postnatal maturation and adult stages are characterized by terminal differentiation and sustained mechanical activity of cardiomyocytes.

### 2.1. Developmental Deployment of A- and B-Type Lamins in the Heart

Nuclear lamins display distinct, cell-type-specific expression patterns during development [23]. In the heart, the nuclear lamina undergoes a coordinated transition from a B-type-dominant state in proliferating embryonic cells to an A-type-enriched state in mature postnatal cardiomyocytes (Figure 1).

Early studies of mouse embryogenesis established that B-type lamins are constitutively expressed from the earliest stages, serving as the foundation of the embryonic nuclear envelope [24,25]. In contrast, lamin A/C expression is developmentally regulated and largely absent during early cleavage and in the inner cell mass [24,25,26,27]. Recent reporter analyses and multi-omics modeling have refined our understanding of its emergence, showing that *Lmna* promoter activity and mRNA levels remain low in pluripotent states but rise sharply at the onset of tissue differentiation (around mouse embryonic day E11), including in the developing heart, outflow tract, and dorsal aorta [28].

This shift in lamin composition becomes most pronounced in the immediate postnatal period. B-type lamins progressively decline, whereas A-type lamins exhibit a reciprocal increase [29,30]. Comparative analyses between mouse E14.5 and postnatal day 0 reveal increased *Lmna* expression alongside reduced *Lmnb1* and *Lmnb2* expression, marking a transition from a B-type-dominant embryonic lamina to an A-type-enriched postnatal nuclear architecture [29,30]. This developmental lamin switch accompanies cardiomyocyte cell-cycle exit and the establishment of long-term contractile function.

### 2.2. Organogenesis: B-Type Lamins Support Nuclear Deformation During Tissue Morphogenesis

Genetic studies indicate that B-type lamins are largely dispensable for early lineage specification but become essential during organogenesis. Pluripotent stem cells lacking both B-type lamins retain the capacity for self-renewal and differentiation into multiple lineages, including cardiomyocytes [30,31,32,33]. Similarly, embryos deficient in lamin B1 or lamin B2 complete early embryogenesis and can even exhibit spontaneous cardiac contraction before perinatal lethality [31,34,35]. These findings indicate that lamin B1 and B2 are not strictly required for establishing lineage identify during the earliest stages of development.

In contrast, B-type lamins deficiency causes profound defects during organogenesis, when cells must undergo coordinated migration, tissue remodeling, and organ assembly. Double-knockout mice lacking both lamin B1 and lamin B2 develop to term but die immediately after birth, displaying severe abnormalities in multiple organs, including the diaphragm and the brain [31]. Likewise, homozygous Lmnb1 or Lmnb2 mutant mice survive embryogenesis but die at birth with reduced body size and defects in lung and skeletal development [31]. These phenotypes highlight an essential role for B-type lamins in supporting tissue morphogenesis during late embryonic development.

A major requirement during organogenesis is the ability of cells to deform and reposition their nuclei within confined tissue environments. In the developing nervous system, lamin B1 and lamin B2 are indispensable for neuronal migration, nucleokinesis, and cortical lamination. Both loss and overexpression of lamin B1 impair nuclear deformation, disrupt neuronal migration, and alter nuclear morphology, demonstrating that B-type lamins regulate nuclear mechanics in a dosage-sensitive manner [31,35,36].

A similar requirement for nuclear remodeling mediated by B-type lamins is observed during cardiac development. Although early heart formation proceeds relatively normally in lamin B1-deficient embryos, later stages of organogenesis reveal pronounced cardiac abnormalities, including hypoplasia of the compact myocardium and defective coronary vessel maturation [37]. These defects arise in part from delayed epicardial cell migration, accompanied by impaired nuclear shape remodeling in vivo and in vitro.

Together, findings from neural and cardiac systems support a conserved role for B-type lamins in enabling nuclear deformation required for cell migration and tissue assembly during organogenesis.

### 2.3. Early Postnatal: Lamin B2 Levels Regulate Cardiomyocyte Proliferation, Polyploidization, and Regenerative Competence

During late embryogenesis, cardiac growth occurs primarily through hyperplasia, with cardiomyocytes dividing to increase cell number [38]. After birth, however, most mammalian cardiomyocytes progressively exit the cell cycle, and subsequent cardiac growth occurs largely through hypertrophic enlargement rather than proliferation [38,39]. This transition marks a fundamental developmental shift from regenerative growth to terminal differentiation.

Consistent with this transition, declining lamin B2 levels mark a critical shift from proliferative competence toward postnatal maturation. lamin B2 is highly expressed during embryogenesis, particularly in cycling cardiomyocytes, but declines sharply after birth, reaching minimal levels by postnatal day 14, coincident with cardiomyocyte cell-cycle withdrawal and progressive polyploidization [30,40]. This developmental decline positions lamin B2 as a key regulator of postnatal cardiomyocyte proliferative capacity.

Loss-of-function studies demonstrate that lamin B2 preserves mitotic competence during cardiac development. Cardiomyocyte-specific Lmnb2 deletion significantly reduces M-phase entry, compromises mitotic fidelity, and promotes karyokinesis failure, leading to polyploidization, a hallmark of cardiomyocyte terminal differentiation [30,41]. These findings directly link lamin B2 to the maintenance of successful mitotic progression during the neonatal period.

Conversely, sustained lamin B2 expression promotes proliferative competence and regenerative potential. lamin B2 overexpression increases mitotic entry, improves completion of cytokinesis, and reduces cardiomyocyte nuclear polyploidy in neonatal hearts. In neonatal mouse models, lamin B2 overexpression enhances myocardial regeneration, whereas *Lmnb2* inactivation impairs regenerative capacity [30]. A similar principle is observed in adult zebrafish, where cardiomyocyte nuclei retain high levels of lamin B2 together with lamin A/C. Sustained lamin B2 expression in zebrafish supports mitotic fidelity and limits polyploidization, suggesting that prolonged B-type lamins expression helps preserve proliferative competence in regenerative species [30].

Importantly, lamin B2 downregulation is not simply associated with loss of proliferative potential but also contributes to cardiomyocyte maturation. Transcriptomic analyses of Lmnb2-deficient hearts reveal activation of maturation-associated gene programs [42]. Similarly, in human induced pluripotent stem cell (hiPSC)-derived cardiomyocytes, LMNB2 loss accelerates sarcomere organization, mitochondrial remodeling, and metabolic maturation while depleting of proliferative cell states [40].

Together, these findings support a model in which lamin B2 functions as a nuclear-envelope-based developmental timer that coordinates cardiomyocyte cell-cycle exit with postnatal maturation. Progressive lamin B2 downregulation links mitotic withdrawal, polyploidization, and activation of maturation programs as part of a coordinated developmental transition from regenerative growth to terminal differentiation.

### 2.4. Postnatal Maturation: Lamin A/C Supports Nuclear Mechanics Under Contractile Load

Following birth, the transition to extrauterine circulation subjects the heart to increasing hemodynamic pressure and continuous contractile load. Under these conditions, lamin A/C becomes increasingly important for reinforcing nuclear stiffness and stabilizing the mature nuclear lamina under load [21]. Although low levels of lamin A/C may contribute to chromatin organization during early developmental stages [40], its principal requirement in vivo emerges during postnatal maturation and long-term mechanical adaptation.

Experimental models strongly support this developmental timing, while recent findings also revealing late embryonic functions of lamin A/C. Animals lacking lamin A/C complete embryogenesis and are born at near-Mendelian ratios [43,44], indicating that lamin A/C is not required for initial heart specification or early cardiac morphogenesis [43,44]. However, recent studies show that *Lmna* deficiency disrupts late embryonic cardiac development mildly, including reduced cardiomyocyte proliferation, premature binucleation, impaired ventricular compaction, and features resembling left ventricular non-compaction (LVNC) [40]. Following birth, lamin A/C-deficient animals rapidly develop severe abnormalities, particularly in mechanically active tissues such as skeletal muscle and the heart [43,44], and multiple *Lmna*-mutant models show progressive structural and functional deterioration during postnatal maturation [29,45,46,47,48].

These findings indicate that the primary developmental role of lamin A/C in the heart lies not in embryonic morphogenesis, but in preserving nuclear stability as cardiomyocytes transition to permanent mechanical function. From this perspective, *LMNA*-associated cardiomyopathy may reflect a failure to maintain the mature nuclear architecture established during maturation.

Viewed in this developmental context, reduced cardiomyocyte proliferation can arise through fundamentally different biological mechanisms. During normal development, progressive lamin B2 downregulation drives an orderly maturation program characterized by cell-cycle exit, polyploidization, and activation of structural and metabolic maturation pathways. In this context, reduced proliferation reflects a physiological transition from regenerative growth to terminal differentiation. By contrast, reduced proliferation in *LMNA*-deficient cardiomyocytes reflects pathological stress-induced terminalization, accompanied by DNA damage, checkpoint activation, and increased vulnerability to mechanical stress. Rather than promoting maturation, lamin A/C dysfunction compromises the ability of postnatal cardiomyocytes to preserve nuclear integrity under load. Together, these observations support a model in which B-type lamins and lamin A/C define distinct nuclear states matched to different developmental stages: one optimized for proliferation and morphogenesis, the other for long-term mechanical resilience.

## 3. Mechanistic Layers of Lamin-Mediated Nuclear Regulation

The developmental roles of lamins described above arise from multiple interconnected molecular mechanisms operating at the nuclear envelope and throughout the nuclear interior. Although lamins were initially described as structural components of the nuclear envelope, they are now recognized as regulators of several key processes that collectively determine nuclear function and cellular behavior. These mechanisms include the spatial organization of chromatin within the nucleus, regulation of mitotic progression and proliferative competence, control of nucleocytoplasmic transport through nuclear pore organization, and transmission of mechanical forces between the cytoskeleton and the genome. Together, these mechanistic layers explain how the nuclear lamina coordinates genome regulation, cell-cycle dynamics, and mechanotransduction during cardiomyocyte development and maturation.

### 3.1. Lamins as Organizers of Cardiac Genome Architecture

Section 2 emphasized the stage-specific deployment of lamin isoforms during cardiac development. The molecular mechanisms discussed below operate across developmental stages and often involve both A- and B-type lamins, whose relative contributions shift as lamin composition changes. Among these mechanisms, regulation of higher-order genome organization is a central function of the nuclear lamina.

The spatial positioning of chromatin within the nucleus is a fundamental component of three-dimensional (3D) genome architecture and is closely linked to transcriptional regulation [49,50,51]. During differentiation, chromatin domains dynamically reposition relative to nuclear structures, including the nuclear lamina, contributing to the four-dimensional (4D) genome in which spatial organization and temporal gene expression are tightly coupled [52]. Within this framework, large chromatin regions termed lamina-associated domains (LADs) occupy the nuclear periphery and are generally enriched for repressive chromatin marks, such as H3K9me2/3 and H3K27me3 [53,54,55]. Because LADs frequently contain developmentally regulated genes lamina association provides a spatial mechanism for transcriptional repression during lineage specification.

Evidence from multiple developmental systems indicates that LAD organization is dynamically remodeled during differentiation [56,57,58,59,60]. During cardiac lineage specification, lamina-mediated chromatin positioning contributes to the temporal control of cardiac gene activation. In embryonic stem cells undergoing cardiogenesis, several cardiac structural and regulatory genes, including *Ttn*, *Actc1*, and *Mef2c* are initially localized within lamin B-associated LADs and remain transcriptionally silent. Upon differentiation, these loci disengage from the nuclear lamina and relocate toward the nuclear interior, coincident with transcriptional activation [61]. Disruption of lamina tethering through Hdac3 or Lap2β results in premature activation of these genes and accelerated differentiation, indicating that LADs positioning functions as a spatial gatekeeper controlling the timing of lineage gene activation. Genome-wide mapping in human adult cardiomyocytes has confirmed that LADs encompass approximately 20% of the cardiomyocyte genome, demonstrating that lamina–chromatin interactions constitute a major architectural layer of the mature cardiac nucleus [62].

Recent genome-wide studies further suggest that the lamina-associated genome is not a uniform repressive compartment but instead exhibits hierarchical organization. Mapping of lamin B1 occupancy across multiple human cell types identified two LAD subclasses, termed T1- and T2-LADs, which differ in lamin association strength, chromatin accessibility, and epigenetic composition [63]. Rather than undergoing abrupt transitions from lamina-associated repression to nuclear interior activation, many developmental genes appear to move through intermediate states during differentiation [64]. For example, the cardiac transcription factor TBX20 transitions from a T1-LAD in embryonic stem cells to a T2-LAD in mesoderm progenitors before relocating to non-LAD regions in cardiomyocytes, where it becomes highly expressed. These observations support a model in which lamina-mediated genome organization regulates cardiac lineage specification through graded spatial remodeling of peripheral chromatin domains.

Peripheral heterochromatin tethering is mediated by multiple factors rather than lamins alone [65,66,67,68,69]. Solovei et al. identified two independent tethers, the lamin B receptor (LBR) and lamin A/C, that anchor heterochromatin to the nuclear periphery [57]. These tethers are deployed sequentially during development: LBR predominates in early stages, whereas lamin A/C expression increases during differentiation and becomes dominant in mature cells. Loss of both LBR and lamin A/C leads to complete detachment of heterochromatin from the nuclear periphery, highlighting their complementary roles [57]. Additional lamin-associated proteins, including PRR14, the cKrox-HDAC3 complex, and multiple LEM-domain proteins such as Emerin, MAN1, Lem2, and LAP family members, function as molecular bridges linking chromatin to the nuclear lamina [67,70,71]. These complexes recruit epigenetic regulators such as histone deacetylase 3 (HDAC3), coupling spatial chromatin positioning to active maintenance of repressive chromatin states through histone deacetylation and heterochromatin formation [72,73].

Beyond LAD positioning, lamins contribute to broader layers of genome organization, including A/B compartment structure, chromosome territory organization, and long-range chromatin interactions [60]. These architectural functions help maintain spatial segregation between transcriptionally inactive chromatin at the nuclear periphery and transcriptionally active regions within the nuclear interior. Complementary structural studies using cryo-electron tomography and cryo-EM have demonstrated that lamin A can directly interact with nucleosomes through a binding motif in its tail domain, providing molecular evidence for direct lamin–chromatin interactions at the nuclear periphery [74].

Lamin-dependent genome organization may also operate earlier in development than previously appreciated. Experiments using lamin triple-knockout mouse embryonic stem cells demonstrate that loss of lamins disrupts LAD positioning and alters higher-order chromatin interactions across the genome [75]. Consistent with this concept, depletion of lamin A/C in stem cells indicates that even low levels of lamin A/C can contribute to tethering lineage-specific genes, including cardiac transcription factors such as *Gata4* and *Gata6*, to the nuclear periphery, thereby preventing premature activation of differentiation programs [40,76]. These findings suggest that lamin-dependent chromatin organization contributes not only to maintenance of differentiated cell identity but also to early lineage restraint during developmental commitment [77].

Consistent with this architectural role, disruption of lamin function perturbs lamin–chromatin interactions in cardiomyocytes. In human iPSC-derived cardiomyocytes carrying *LMNA* mutations, specific subsets of lamina-associated chromatin regions lose peripheral association and exhibit increased transcriptional activity [78]. These regions are enriched for genes associated with non-myocyte lineages, suggesting that defective lamina tethering may permit inappropriate activation of alternative lineage programs during cardiac differentiation. Similar lineage instability has been observed in skeletal muscle, where *LMNA* depletion represses of myogenic transcription factors while activating neural lineage genes [79]. Together, these findings support a model in which the nuclear lamina safeguards cardiomyocyte identity by coupling spatial genome organization to stable transcriptional repression. Disruption of this architectural framework permits lineage-inappropriate gene activation and provides a mechanistic link between lamin dysfunction, developmental instability, and cardiomyopathy.

### 3.2. Lamins in Mitotic Control and Proliferative Competence

In addition to organizing interphase genome architecture, lamins also play important roles during mitosis, linking nuclear structure to the regulation of proliferative competence. During cell division, the nuclear lamina undergoes extensive remodeling to permit nuclear envelope breakdown (NEB), spindle assembly, and accurate chromosome segregation. Entry into mitosis triggers phosphorylation-dependent disassembly of lamin polymers, allowing lamina depolymerization and nuclear envelope disassembly [3]. Disruption of lamin function therefore has the potential to impair mitotic fidelity and compromise proliferative competence, thereby influencing regenerative capacity [80,81].

Consistent with the role of lamins in supporting mitosis, B-type lamins contribute to spindle organization and mitotic architecture. Several studies suggest that B-type lamins participate in the spindle matrix and help regulate microtubule assembly, thereby supporting proper spindle architecture during mitosis [30,80,82,83]. Perturbation of lamin function disrupts spindle morphology, spindle orientation, and chromosome segregation, highlighting the importance of lamins in maintaining mitotic fidelity [14]. These observations support the concept that lamins contribute to the mechanical stability of the mitotic apparatus [14,30,80,82,83].

Mechanistically, lamin B2 deficiency impairs nuclear envelope breakdown during prometaphase. Nuclear envelope breakdown is a tightly coordinated process involving lamina depolymerization, nuclear membrane remodeling, and spindle microtubule penetration into the nuclear space [84]. Proper NEB is required to allow spindle microtubules to access chromosomes and establish kinetochore attachments [85]. When lamin B2 is absent, spindle access to chromosomes becomes limited, weakening microtubule–centromere interactions and reducing recruitment of chromosomal passenger proteins such as Aurora B kinase [30,80,86]. These defects ultimately compromise metaphase progression and completion of karyokinesis, leading to mitotic failure and polyploidization in cardiomyocytes.

Beyond its structural role in mitosis, lamin B2 also influences upstream cell-cycle regulatory pathways that control mitotic entry. In cardiomyocytes, *Lmnb2* deficiency is associated with reduced Cyclin B1 protein abundance and decreased Cyclin B1 phosphorylation, indicative of impaired activation of the G2/M transition [30]. Consistent with a broader role in proliferative control, lamin B2 promotes proliferation in several cancer types, including colorectal and bladder cancers, through regulation of cell-cycle pathways involving p21 and the cell-division regulator CDCA3 [87,88]. These findings suggest that B-type lamins contribute not only to structural events during mitosis but also to signaling pathways that sustain proliferative competence.

Together, these studies indicate that lamins regulate multiple stages of mitotic progression, from nuclear envelope remodeling and spindle assembly to cell-cycle entry. In cardiomyocytes, where proliferative capacity becomes progressively restricted during development, perturbation of these lamin-dependent processes can directly influence the transition between proliferative growth and terminal differentiation and consequently impact cardiac regenerative potential.

### 3.3. Lamins, Nuclear Pore Organization, and Transcriptional Responsiveness

Beyond their roles in genome architecture and mitotic control, lamins also influence gene regulation by organizing nuclear pore complexes (NPCs) and modulating nucleocytoplasmic transport. NPCs are large macromolecular assemblies embedded in the nuclear envelope that mediate the selective exchange of proteins, RNA, and signaling molecules between the nucleus and cytoplasm [89,90,91]. Because many transcription factors and signaling mediators require regulated nuclear import and export, the abundance and spatial organization of NPCs can strongly influence the transcriptional responsiveness of a cell.

Nuclear lamina plays an important role in stabilizing NPC distribution and maintaining nuclear envelope architecture. Lamins interact with nucleoporins and nuclear envelope components that regulate NPC positioning and stability within the nuclear membrane [92,93]. Structural and biochemical studies demonstrate that lamins interact with multiple nucleoporins and contribute to proper NPC positioning within the nuclear envelope [92,94]. Disruption of lamins can alter nuclear pore density and spatial distribution, leading to clustering of NPCs or regions of reduced pore abundance. Such changes in NPC organization can in turn affect nucleocytoplasmic transport efficiency and downstream gene regulatory programs.

Emerging evidence suggests that B-type lamins modulate the transcriptional capacity of differentiated cells through this regulatory axis [42,95,96]. For example, in postnatal mouse hearts lamin B2 downregulation markedly decreases nuclear pore numbers [42]. Reduced NPC abundance may limit nucleocytoplasmic transport efficiency and dampen the magnitude of stress-responsive transcriptional responses during cardiac remodeling [97,98]. Consistent with this idea, alterations in nuclear pore organization can constrain the ability of cardiomyocytes to rapidly activate transcriptional programs required for adaptive responses to physiological stress [94,99].

Similar mechanisms have been observed in other differentiated cell types. In olfactory sensory neurons, lamin B1 deficiency causes clustering of nuclear pore complexes and impairs the activation of mature neuronal gene programs, despite otherwise normal early differentiation [96]. These observations suggest that lamins contribute to the spatial organization of NPCs and thereby influence the efficiency of nuclear transport pathways that regulate transcriptional activation.

More broadly, the nuclear lamina is increasingly recognized as a structural scaffold that coordinates nuclear transport capacity with cell-state transitions. By regulating NPC abundance and distribution, lamins can influence the rate at which transcription factors, chromatin regulators, and RNA species shuttle across the nuclear envelope [100,101,102]. Through this mechanism, lamin-dependent NPC organization may help tune the amplitude and timing of gene regulatory signals during developmental maturation and cellular adaptation.

Together, these findings support a model in which lamins regulate transcriptional responsiveness not only through direct chromatin organization but also through control of nuclear pore architecture and nucleocytoplasmic transport capacity. This regulatory layer may be particularly important in cardiomyocytes, where rapid transcriptional adaptation to mechanical and metabolic stress is required to maintain long-term cardiac function. Thus, lamins influence transcription not only by organizing chromatin at the nuclear periphery but also by controlling the nuclear transport pathways that regulate transcription factor access to the genome.

### 3.4. Lamins Integrate Nucleo–Cytoskeletal Mechanics, Metabolism, and Epigenetic Stability

In addition to organizing genome architecture and mitotic progression, lamins function as central components of the nucleo–cytoskeletal force-transmission system that links extracellular and cytoskeletal forces to nuclear structure and gene regulation. Mechanical forces are fundamental regulators of cellular behavior, and the cytoskeleton–nucleus axis acts as a key mediator of cellular responses to physical cues from the extracellular environment [103]. Forces generated by the cytoskeleton are transmitted to the nucleus through the Linker of Nucleoskeleton and Cytoskeleton (LINC) complex and associated inner nuclear membrane proteins, placing the nuclear lamina at the interface between cytoskeletal tension and nuclear integrity [104,105]. Through interactions with both A- and B-type lamins, the LINC complex mechanically couples cytoskeletal networks to the nuclear envelope, enabling cytoplasmic forces to influence nuclear structure and signaling [106,107].

During early development, B-type lamins are particularly important in this system because they form a loosely organized, deformable lamin B1 filament network beneath the inner nuclear membrane [108,109]. Coupled to the cytoskeleton via the LINC complex, this network facilitates force transmission and dissipation while preserving nuclear integrity. This mechanical adaptability enables reversible nuclear deformation and nuclear plasticity required for cell migration and tissue morphogenesis [104,105,110,111]. Studies in endothelial cells demonstrate that Nesprin-1 and Nesprin-2 regulate nuclear architecture and influence endothelial cell migration and angiogenic loop formation, whereas Nesprin-3 contributes to flow-induced centrosome polarization and directional migration [112,113]. These observations support a role for B-type lamins in maintaining nuclear plasticity and mechanical resilience during developmental processes that require extensive cell movement.

In mechanically active tissues such as the heart and skeletal muscle, lamin A/C assumes a particularly important role in this force-transmission system [114,115]. By reinforcing the nuclear lamina and stabilizing cytoskeletal connections, lamin A/C enables the nucleus to withstand sustained mechanical stress generated by repeated contractile activity and serves as a key mediator of nuclear mechanotransduction linking cytoskeletal forces to chromatin organization and transcriptional regulation [116,117]. However, recent work in cardiomyocytes has refined this model of nuclear force transmission. Contrary to earlier assumptions that the LINC complex is the primary conduit for contractile forces, experimental evidence indicates that the perinuclear microtubule network is a dominant driver of nuclear deformation and damage in *LMNA*-deficient cardiomyocytes [118]. Disrupting this microtubule cage, rather than perturbing the LINC complex itself, prevents nuclear rupture and improves cardiac function in *LMNA* cardiomyopathy models, highlighting a critical role for microtubule-derived forces in cardiac nuclear pathology [118]. These findings suggest that the relative contributions of different cytoskeletal systems to nuclear mechanics are tissue-specific and may be particularly influenced by the unique contractile architecture of cardiomyocytes.

Lamins are also major determinants of nuclear mechanical properties. Experimental studies demonstrate that lamin composition strongly influences nuclear stiffness, with lamin deficiency producing mechanically fragile nuclei that deform readily under applied stress [114,119]. Importantly, nuclear mechanics arise not only from the lamina itself but also from interactions between lamins and chromatin. Chromatin contributes substantially to nuclear rigidity, and interactions between lamins and peripheral heterochromatin help stabilize nuclear structure under mechanical strain [84,120]. Consistent with these observations, lamin A levels scale with tissue stiffness across diverse cell types and promote matrix-directed differentiation programs [23]. These findings support a model in which lamin composition adapts to the mechanical properties of the cellular microenvironment, thereby linking tissue mechanics to nuclear architecture and gene regulation.

Beyond mechanical force transmission, lamin-dependent mechanotransduction also influences intracellular metabolic and epigenetic states. Recent studies demonstrate that lamin A/C directly regulates metabolic pathways that control chromatin modification. In particular, lamin A/C represses the expression of cysteine-metabolizing enzymes such as CTH and CBS, thereby limiting cysteine flux into acetyl-CoA production. Loss of lamin A/C releases this repression, increasing acetyl-CoA availability and driving hyperacetylation of histone residues including H3K9ac and H3K27ac [121]. This metabolic–epigenetic coupling can alter lineage stability and cellular identity, positioning cellular metabolism as a key downstream effector of lamin function [121]. In parallel, lamin A/C deficiency induces metabolic remodeling characterized by activation of AMPK signaling and altered lipid metabolism, which further impairs differentiation and cellular homeostasis [122].

Consistent with this integrative role, lamin dysfunction also disrupts epigenetic stability under mechanical stress. In mechanically challenged muscle cells lacking lamin A/C, promoter accessibility increases and chromatin modifications shift toward active states, including elevated H3K4me3 and reduced H3K27me3, accompanied by altered histone deacetylation and DNA methylation pathways [123]. Application of cyclic mechanical strain further amplifies these chromatin changes in lamin-deficient cells, demonstrating that mechanical stress is actively transduced into altered epigenetic states when nuclear structural integrity is compromised [123]. These findings highlight how lamins normally buffer mechanical inputs to preserve transcriptional programs required for stable cellular identity.

Taken together, these observations indicate that lamins function as integrative regulators of nuclear physiology. By coordinating genome organization, mitotic progression, nucleocytoplasmic transport, and mechanotransduction, lamins align nuclear architecture with cellular state and environmental demands. In the heart, where cardiomyocytes transition from proliferative embryonic cells to long-lived contractile cells exposed to continuous mechanical stress, this integrative role becomes particularly critical. Disruption of lamin-dependent regulatory networks can propagate across multiple nuclear processes, including mechanical stability, chromatin organization, metabolism, and epigenetic regulation, providing a mechanistic framework for the diverse cardiac phenotypes associated with lamin dysfunction. These interconnected mechanisms also help explain why mutations in lamins and lamin-associated proteins give rise to a broad spectrum of tissue-specific diseases, particularly in mechanically active organs such as the heart.

## 4. Lamins in Cardiac Disease and Aging

The differential developmental roles of lamin classes provide a useful framework for understanding their divergent disease associations. Mutations in lamins and lamin-associated proteins cause a broad spectrum of disorders, collectively termed laminopathies, that affect striated muscle, adipose tissue, peripheral nerve, skin, and systemic aging pathways. Representative examples include Emery-Dreifuss muscular dystrophy (EDMD), characterized by skeletal muscle weakness, joint contractures, and cardiac conduction disease; Hutchinson–Gilford progeria syndrome (HGPS), a premature aging disorder caused by abnormal lamin A processing; and familial partial lipodystrophy (FPLD), which highlights the role of lamins in adipose tissue identity and metabolic homeostasis. Together, these disorders demonstrate that lamins function not only as structural components of the nuclear envelope but also as key regulators of genome organization, tissue integrity, and long-term cellular homeostasis across multiple organ systems.

### 4.1. LMNA-Related Cardiomyopathy

Among laminopathies, *LMNA* mutations produce one of the most clinically diverse disease spectra, affecting skeletal muscle, adipose tissue, peripheral nerve, and systemic aging pathways. Despite this broad phenotypic range, cardiac involvement is among the most frequent and life-threatening manifestations of *LMNA* disease.

*LMNA*-related cardiomyopathy is one of the most clinically important forms of inherited dilated cardiomyopathy (DCM), distinguished by a high burden of conduction disease, arrhythmia, heart failure, and sudden cardiac death. A striking feature of this disorder is that electrical abnormalities often emerge before advanced ventricular remodeling. In several reported families, variants such as E82K, S143P, and R189W were associated with atrioventricular block, atrial fibrillation, ventricular arrhythmias, and progressive dilated cardiomyopathy, illustrating that electrical instability often precedes advanced ventricular remodeling [124,125,126]. At the same time, the clinical spectrum is broader than a uniform conduction-plus-DCM phenotype. For example, the P509Lfs*39 frameshift variant was associated with giant right atrium, sick sinus syndrome, atrial and ventricular arrhythmias, and progressive atrial standstill, suggesting that some *LMNA* variants may produce an atrial-predominant electrical cardiomyopathy [127]. Pathogenicity is also not limited to missense or truncating variants: the synonymous variant c.936G>A (p.Gln312Gln) disrupts splicing, reduces *LMNA* expression, and associates with dilated cardiomyopathy, underscoring the importance of variant-specific interpretation in *LMNA* disease [128].

A major challenge in the field is that *LMNA*-related cardiomyopathy cannot be explained by a single linear mechanism. Instead, current evidence supports a multilayered pathogenic cascade in which primary defects in nuclear lamina integrity and genome organization trigger secondary abnormalities in organelle homeostasis, stress signaling, and electrophysiological regulation. These layers are partially shared across variants but are engaged to different degrees depending on mutation type, cellular context, and mechanical demand. This framework helps explain how diverse *LMNA* mutations converge on a shared clinical spectrum of conduction disease, arrhythmia, fibrosis, and progressive ventricular dysfunction while still producing substantial mutation-specific heterogeneity. It also provides the basis for emerging mechanism-guided rescue strategies and highlights the need for variant-informed precision approaches in *LMNA* cardiomyopathy. The major mechanistic frameworks and representative rescue studies discussed below are summarized in Table 1.

#### 4.1.1. Primary Nuclear Defects: Structural Destabilization and Chromatin Dysregulation

The nuclear mechanics model remains a foundational framework for understanding *LMNA* cardiomyopathy. In this view, *LMNA* mutations weaken the mechanical stability of the nuclear lamina, rendering cardiomyocyte nuclei more susceptible to deformation and damage under repetitive contractile stress. Early support for this model came from Lmna-deficient mice, in which loss of lamin A/C caused nuclear shape abnormalities, heterochromatin disruption, detachment of desmin filaments from the nuclear surface, and defective nucleo–cytoskeletal force transmission, ultimately leading to dilated cardiomyopathy [44]. The functional importance of these structural defects was reinforced by cardiomyocyte-specific re-expression of lamin A, which partially restored desmin and connexin-43 localization and improved cardiac function [129]. Likewise, lamin A/C haploinsufficiency was sufficient to disrupt nuclear architecture and induce early apoptosis of atrioventricular nodal myocytes before overt cardiomyopathy developed [47].

Disease-associated missense and nonsense variants further support this model. *Lmna* H222P/H222P mice develop a striated muscle laminopathy phenotype with muscular dystrophy, conduction abnormalities, fibrosis, and dilated cardiomyopathy [45]. The E82K rod-domain mutation likewise disrupts lamin assembly and nuclear envelope integrity and triggers extensive cardiomyocyte apoptosis and fibrosis [149]. In the N195K model, chamber dilation, conduction defects, fibrosis, and abnormal connexin localization suggest that structural perturbation of the lamina can propagate outward to cytoskeletal and membrane-associated systems involved in coordinated contraction and conduction [46]. Similarly, the R225X knock-in model links *LMNA* haploinsufficiency to extracellular matrix accumulation, fibrosis, myocardial stiffness, and impaired electrical conduction, extending the mechanical model from nuclear instability to tissue-level electromechanical dysfunction [48]. Even the historically designated *Lmna*^−/−^ model, later reinterpreted as an *Lmna*Δ8-11 allele expressing truncated lamin A Δ8-11 rather than a true null, retained severe nuclear envelope deformation, heterochromatin loss, and emerin mislocalization, reinforcing the centrality of lamin-dependent nuclear architecture in disease [43,150]. Importantly, a CRISPR-engineered primate model carrying the *LMNA* c.357-2A>G splice mutation developed arrhythmia, dilated cardiomyopathy, fibrosis, nuclear malformation, and cardiomyocyte disarray, supporting the translational relevance of nuclear structural disruption beyond rodent systems [136].

However, nuclear fragility alone does not fully explain the diversity of *LMNA* phenotypes. Lamin A/C also functions as a major organizer of peripheral chromatin and lineage-specific transcriptional programs, and growing evidence indicates that disruption of this role contributes directly to cardiomyopathy. In patient-derived hiPSC-derived cardiomyocytes (hiPSC-CMs) carrying the T10I and R541C variants, mutant cells lose lamin B1 occupancy at specific LADs and aberrantly activate non-cardiac lineage genes, indicating a failure to maintain cardiomyocyte identity [78]. Similarly, loss of lamin A/C in mouse embryonic stem cells and embryos results in premature activation of cardiac developmental genes such as *Gata4*, leading to precocious cardiomyocyte differentiation and abnormal cardiac phenotypes cells [40]. Single-cell analysis of the c.357-2A>G splice-site mutation further showed lineage-specific transcriptional dysregulation affecting both cardiomyocyte and epicardium-derived trajectories in the setting of *LMNA* haploinsufficiency [133]. Consistent with this, chromatin conformation studies in a haploinsufficient hiPSC-CM model showed altered A/B compartment dynamics and ectopic activation of non-cardiac transcriptional programs, further supporting the idea that lamin A/C deficiency perturbs higher-order genome organization in cardiomyocytes [130]. Additional support comes from the K117fs iPSC-CM model, in which chromatin dysregulation was associated with abnormal gene expression and secondary activation of the PDGF pathway [131], and from the R541C mouse model, where increased heterochromatin marks were linked to mitochondrial dysfunction [151]. The synonymous c.936G>A (p.Gln312Gln) variant further broadens this framework because aberrant RNA splicing and nonsense-mediated decay reduce *LMNA* dosage and likely compromise its chromatin-organizing functions [128]. At a more network-centered level, the Q353R model showed that mutant lamin A/C traps TEAD1 at the nuclear membrane, disrupting Hippo/YAP-TEAD-dependent transcription and impairing cardiomyocyte structural maturation [132].

These structural and chromatin-based mechanisms should therefore be viewed as interdependent manifestations of nuclear dysfunction. Altered lamina architecture weakens both mechanical resilience and genome regulation, predisposing cardiomyocytes to downstream injury. Notably, several pathogenic features of *LMNA* cardiomyopathy, particularly transcriptional misregulation and altered lineage restraint, echo developmental functions of lamin A/C discussed earlier in this review.

#### 4.1.2. Secondary Disease Amplifiers: Stress Signaling and Organelle Homeostasis

Downstream of these primary nuclear defects, *LMNA*-mutant cardiomyocytes activate a broad network of stress-response pathways that amplify disease progression. These include DNA damage signaling, inflammatory pathways, MAPK activation, endoplasmic reticulum stress, mitochondrial dysfunction, and apoptosis. In most cases, these pathways function as secondary but pathogenic amplifiers rather than primary initiating mechanisms.

Evidence for this concept is particularly strong for DNA damage signaling. In a D300N transgenic mouse model, mutant lamin A expression activated an E2F-DDR signaling axis, leading to fibrosis, apoptosis, and severe dilated cardiomyopathy; partial rescue by cardiac-specific deletion of *Tp53* demonstrated that DNA damage signaling contributes causally to disease severity [134]. Similarly, cardiomyocyte-specific *LMNA* deficiency activated the cGAS-STING inflammatory pathway, promoting fibrosis and progressive cardiac dysfunction; genetic ablation of cGAS improved survival and cardiac function [135].

Stress-activated kinase signaling represents another major disease amplifier. In the H222P *LMNA* model, ERK1/2 activation occurred before overt cardiomyopathy and promoted sarcomeric disorganization and ventricular dysfunction; pharmacologic ERK1/2 inhibition improved cardiac performance [139]. Additional studies have shown that the p38α branch of MAPK signaling is also activated early in *LMNA* cardiomyopathy, and pharmacologic inhibition prevented ventricular dilation and improved cardiac function in mouse models [137]. Likewise, in patient-derived iPSC-CMs carrying the R225X mutation, electrical stimulation induced MEK1/ERK1/2 activation, increased nuclear abnormalities, and promoted apoptosis, whereas treatment with U0126 or selumetinib reduced these defects [138].

Organelle-centered stress responses add a further layer of disease amplification. In cardiomyocytes expressing the truncated R321X variant, mislocalized lamin A/C accumulated in the endoplasmic reticulum and triggered a PERK-dependent unfolded protein response (UPR), calcium dysregulation, and apoptosis signaling [140]. Proteomic and transcriptomic studies across multiple *LMNA* variants similarly reveal convergent disturbances in mitochondrial metabolism, oxidative stress, extracellular matrix remodeling, and ion-channel pathways [141,142,152]. Together, these findings suggest that diverse *LMNA* mutations converge on a shared state of impaired intracellular stress adaptation.

#### 4.1.3. Electrophysiological Remodeling and Arrhythmogenesis

The early clinical prominence of conduction disease and arrhythmia indicates that electrophysiological dysfunction is a core feature of *LMNA* cardiomyopathy rather than a late consequence of structural remodeling. Current evidence suggests that *LMNA* mutations impair cardiac excitability through a combination of ion-channel dysregulation, altered intercalated-disk signaling, and abnormal calcium handling.

One major branch of this model involves sodium channel dysfunction. In *Lmna* N195K/N195K ventricular myocytes, prolonged action potentials, increased early and delayed afterdepolarizations, triggered arrhythmic activity, and increased peak and late sodium current indicate that abnormal sodium handling contributes directly to arrhythmogenesis [143]. Complementary evidence comes from the K219T patient-specific iPSC-CM model, in which reduced excitability and slower conduction velocity were linked to repression of SCN5A and reduced Nav1.5-mediated sodium current [144]. In the R249Q iPSC-CM model, reduced sodium current density and altered sodium current kinetics were accompanied by dysregulation of PKP2, DSP, and GJA5, suggesting that *LMNA* variants may impair conduction both directly through sodium channel regulation and indirectly through altered intercalated disk organization [145,146].

Calcium handling provides a second major route to arrhythmia vulnerability. In S143P hiPSC-CMs, mutant cells displayed bradyarrhythmia, severe arrhythmic beating, and abnormal calcium handling together with activation of ER stress and MAPK signaling, suggesting that electrical instability can arise from an impaired ability to maintain calcium homeostasis under stress [146]. This concept is reinforced by a stretch-based hiPSC-CM model of *LMNA*-related DCM, in which mutant cardiomyocytes showed exaggerated mechanically induced Ca^2+^ influx mediated primarily by TRPV4, with additional contribution from TRPV2 [147]. Pharmacological inhibition of TRPV4 markedly reduced the stretch-induced Ca^2+^ response, linking mechanical vulnerability of *LMNA*-mutant cardiomyocytes to downstream arrhythmogenic calcium overload.

Taken together, these studies indicate that electrophysiological dysfunction in *LMNA* cardiomyopathy can arise through both direct ion channel dysregulation and broader disruption of the structural and signaling context required for normal excitability. This helps explain why conduction disease and arrhythmia often appear early, sometimes before advanced structural remodeling is evident.

#### 4.1.4. Precision Therapy and Mechanism-Guided Rescue

Current treatment of *LMNA*-related cardiomyopathy remains primarily supportive rather than disease-modifying. Clinical management of *LMNA*-related cardiomyopathy is still focused on prevention and treatment of conduction disease, atrial and ventricular arrhythmias, and heart failure progression, using standard heart-failure pharmacotherapy together with device-based and rhythm-directed interventions when indicated. Because *LMNA* cardiomyopathy carries a relatively high arrhythmic risk, diagnosis has direct implications for surveillance and implantable cardioverter–defibrillator (ICD) decision-making, in addition to family screening and genetic counseling. Advanced therapies, including heart transplantation, remain necessary in a subset of patients with progressive disease. Therefore, although current care improves risk management and clinical outcomes, it is not yet truly disease-modifying, which provides the rationale for mechanism-guided and variant-informed rescue strategies [153,154].

Against this background, the mechanistic heterogeneity of *LMNA* cardiomyopathy has important therapeutic implications: different variants are unlikely to respond uniformly to a single treatment strategy. Instead, rescue studies increasingly suggest that distinct pathogenic pathways may represent variant-specific therapeutic targets.

One class of rescue studies has targeted pathway-level disease amplifiers. In the K117fs iPSC-CM model, chromatin dysregulation and secondary activation of PDGF signaling were associated with arrhythmic and electrophysiological abnormalities, and PDGFRB inhibition with agents such as sunitinib, sorafenib, or axitinib ameliorated the phenotype [131]. In the H222P model, inhibition of ERK1/2 improved sarcomeric organization and cardiac function, supporting stress kinase signaling as a tractable intervention point [139]. In cardiomyocyte-specific *LMNA* deficiency, interruption of cGAS-STING signaling improved survival and cardiac performance, identifying inflammatory sensing of nuclear damage as another actionable pathway [135]. Likewise, in the Q353R model, disruption of Hippo/YAP-TEAD signaling was linked to therapeutic responsiveness to TT-10 [132]. In the R321X model, salubrinal, guanabenz, and empagliflozin alleviated PERK/UPR-dependent ER stress and improved calcium handling and pro-survival signaling [140]. Together, these studies suggest that distinct *LMNA* variants may generate mutation-specific “druggable nodes” across chromatin-linked signaling, stress kinase activation, inflammatory sensing, and organelle stress pathways.

A second group of rescue strategies targets electrophysiological effectors. In *Lmna* N195K/N195K mouse myocytes, suppression of late sodium current with ranolazine or low-dose TTX reduced triggered activity and shortened action potential duration [143]. In the K219T patient-specific iPSC-CM model, restoration of SCN5A/Nav1.5 function rescued reduced excitability and conduction slowing [144]. These studies show that, in some contexts, meaningful rescue can be achieved not by directly correcting the nuclear defects but by targeting the final electrophysiological outputs through which the *LMNA* mutation compromises cardiomyocyte function.

A third and especially informative category involves variant-specific transcripts or protein rescue. In patient fibroblasts and hiPSC-CMs carrying R225X, Q354X, or T518fs, treatment with PTC124 restored lamin A/C expression and improved cellular function in the R225X setting, but not in Q354X or T518fs, despite all three being truncating variants [148]. This result is particularly important because it shows that even superficially similar classes may differ substantially in therapeutic responsiveness, likely owing to differences in stop codon context, transcript stability, and nonsense-mediated decay. In that sense, *LMNA* cardiomyopathy provides a compelling example of why variant-level, rather than purely gene-level, precision medicine may be necessary.

Together, these studies support the concept that *LMNA*-related cardiomyopathy is amenable to mechanism-guided therapeutic intervention, although variant-specific stratification will likely be required. The p38α inhibitor PF-07265803 advanced to phase 3 clinical testing in the REALM-DCM trial, marking an important milestone for mechanism-based therapy in *LMNA* disease, even though the trial ultimately failed to demonstrate clinical efficacy [155]. These experiences highlight both the promise and the challenges of translating mechanistic insights from *LMNA* disease models into clinical benefit.

Notably, many pathogenic mechanisms described in *LMNA* cardiomyopathy reflect failure of the normal developmental functions of lamin A/C discussed earlier in this review, including its roles in postnatal mechanical adaptation, chromatin organization, and stress-responsive gene regulation.

### 4.2. B-Type Lamins in Disease

Although B-type lamins play critical roles during embryonic cardiac development, human diseases caused by B-type lamins dysfunction rarely present as primary cardiomyopathy. This contrasts sharply with *LMNA* mutations, which are a major cause of inherited dilated cardiomyopathy and conduction disease. One explanation lies in developmental timing: B-type lamins are most essential during embryogenesis, where they support nuclear plasticity, cell migration, mitotic fidelity, and tissue morphogenesis, whereas lamin A/C becomes increasingly important after birth as cardiomyocytes transition to permanent mechanical function. Severe loss of B-type lamins is often embryonic or perinatally lethal, limiting the opportunity for postnatal cardiac disease to manifest. In addition, partial functional compensation between lamin B1 and lamin B2, as well as by other nuclear envelope components, may buffer cardiac-specific phenotypes. As a result, B-type lamins dysfunction more commonly manifests in tissues that depend on persistent nuclear remodeling and lineage maintenance, particularly the nervous system.

The best characterized B-type lamins disorder is adult-onset autosomal dominant leukodystrophy (ADLD), caused by *LMNB1* gene duplication and marked by progressive central nervous system demyelination, autonomic dysfunction, cerebellar ataxia, and cognitive decline. Mechanistically, lamin B1 overexpression disrupts chromatin organization, nuclear pore function, and oligodendrocyte differentiation, leading to impaired myelin gene expression and altered lipid metabolism in the CNS. Patient-derived iPSC cerebral organoids further show that elevated lamin B1 directly impairs human neuronal migration: ADLD neurons exhibit increased lamin B1 levels, increased nuclear stiffness, and defective radial migration within a three-dimensional tissue environment [36].

Beyond ADLD, lamin B dysregulation has been implicated in a broader spectrum of neurodevelopmental and neurodegenerative disorders. These include *LMNB2*-associated progressive myoclonus epilepsy with early ataxia [156,157], as well as conditions linked to defective nuclear envelope integrity, impaired neuronal migration, and abnormal neural tube closure. Reduced lamin B levels have also been implicated in age-associated neurodegenerative settings such as Alzheimer’s disease, where lamina disruption is associated with chromatin relaxation and neuronal vulnerability. In addition to neurological phenotypes, rare *LMNB2* mutations have also been reported in patients with partial lipodystrophy, suggesting that B-type lamins may contribute to metabolic tissue dysfunction and adipose tissue homeostasis [158,159]. Collectively, these findings highlight a central role for B-type lamins in brain development, neuronal nuclear dynamics, and glial function, while their contribution to cardiac disease remains far less well defined.

A recent high-throughput chemical screening study in ADLD models showed that lamin B1 protein levels can be reduced by non-toxic, brain-penetrant small molecules, providing proof-of-principle that B-type lamins are pharmacologically modifiable [160,161]. Although these findings derive from neurological systems, they raise the possibility that lamin B dysregulation could become therapeutically tractable in other tissue contexts as well. More broadly, current evidence suggests that whereas A-type lamins dysfunction preferentially manifests in mechanically stressed tissues such as the heart and skeletal muscle, B-type lamins dysfunction more often affects systems that depend on nuclear plasticity, migration, and lineage control, particularly in the nervous system.

### 4.3. Lamins and Cellular Senescence in Cardiovascular Tissues

Beyond inherited laminopathies, age-associated changes in lamina composition, particularly loss of lamin B1, have emerged as important features of cellular senescence in cardiovascular tissues [162,163]. Reduction in lamin B1 is a well-established hallmark of senescence across multiple cell types, suggesting that lamina remodeling may represent a convergent link between aging, genome instability, and altered tissue homeostasis.

Recent work has extended this concept to cardiovascular cell types. Chemical and mechanical stimuli can induce senescence in primary murine cardiac fibroblasts through distinct upstream pathways, yet both converge on common downstream features including reduced lamin B levels and cell-cycle arrest, indicating that nuclear lamina remodeling is a shared hallmark of the senescent state [164]. By contrast, genetic ablation of lamin A/C in cardiac fibroblasts does not robustly enhance classical senescence markers, whereas mutant lamin B1 in embryonic fibroblasts produces marked nuclear shape abnormalities, impaired differentiation, polyploidization, and premature senescence [165]. Together, these observations suggest that lamin B may be a more functionally relevant determinant of senescence in fibroblast-like mesenchymal cells.

This concept is also consistent with aging studies in other systems. In *Drosophila*, lamin B gradually declines in aging fat body cells, whereas lamin C remains relatively stable [166], supporting the idea that reduction in B-type lamins is more closely linked to age-associated lamina remodeling than changes in A-type lamins. Although these findings come from non-cardiac systems, they reinforce a broader principle that lamin B loss may be a recurring feature of senescent and aging cells.

From the perspective of cardiovascular disease, these observations raise the possibility that lamina remodeling links inherited lamin dysfunction, cellular aging, and chronic tissue decline through partially overlapping mechanisms. Although the senescence field in the heart remains less developed than the laminopathy field, emerging evidence suggests that alterations in lamina composition, especially lamin B loss, may contribute to age-associated fibroblast dysfunction, impaired stress adaptation, and progressive remodeling in cardiovascular tissues. More broadly, these findings suggest that age-related changes in lamina composition may represent a convergence point between developmental lamin functions, cellular senescence, and chronic tissue decline.

## 5. Conclusions and Future Perspectives

The coordinated deployment of nuclear lamins serves as a fundamental hallmark of cardiac maturation. As detailed in this review, the nuclear lamina undergoes dynamic remodeling, transitioning from a B-type-dominant state in early embryogenesis to an A-type-enriched state following the onset of tissue differentiation. This shift, which intensifies through late embryogenesis and into the early postnatal period, parallels the critical transition of cardiomyocytes from a proliferative state to one defined by terminal differentiation and sustained mechanical activity. Despite these insights, several questions remain regarding the “lamin code” in both cardiac health and broader tissue development:

**Mechanosensitive Thresholds:** While lamin composition evolves to meet the increasing mechanical demands of the postnatal heart, it remains to be fully determined at what point specific variants or imbalances in the lamina disrupt nuclear integrity under chronic cyclic stress. Understanding how these alterations perturb the nuclear-cytoskeletal linkage established during late organogenesis may explain the clinical heterogeneity seen across various lamin-related pathologies.

**The Regenerative Barrier:** The transition from B-type to A-type lamins in mammals coincides closely with the loss of cardiac regenerative capacity. This suggests that the transition of the nuclear lamina might act as a physical or epigenetic gatekeeper for cell-cycle reentry. Exploring whether a more plastic, “embryonic-like” nuclear profile can be transiently re-established could potentially unlock new therapeutic pathways for tissue regeneration.

**3D Genome Stability:** Beyond providing structural support, lamins are essential for organizing chromatin and stabilizing lineage commitment. High-resolution mapping of lamina-associated domains (LADs) during developmental transitions may clarify how lamin-dependent organization prevents the transcriptional “drift” often observed in age-related or genetic diseases.

Moving forward, the challenge lies in developing interventions that address the primary biophysical and regulatory defects inherent in a compromised nuclear lamina. By identifying the specific points where the developmental “lamin code” goes awry, we can move toward precision strategies that reinforce the nucleus against mechanical stress while stabilizing the transcriptional landscape. Furthermore, understanding the transition from a plastic, B-type-dominant embryonic state to a mature, A-type-enriched lamina may offer a transformative perspective on cardiac repair. If the maturation of the nuclear lamina indeed serves as a gatekeeper for cell-cycle exit, transiently modulating these structural constraints could provide a novel pathway to both prevent disease progression and stimulate endogenous regeneration, ensuring long-term functional integrity in the face of genetic or age-related challenges.

## Figures and Tables

**Table 1:** **Mechanistic frameworks of *LMNA*-related cardiomyopathy**. *LMNA*-related cardiomyopathy arises through partially overlapping pathogenic layers rather than a single linear mechanism. The table summarizes representative models, shared phenotypes, dominant mechanisms, and proof-of-principle rescue strategies relevant to this framework.

Pathogenic Framework	Representative Variants/Models	Shared Cardiac Phenotypes	Core Mechanism	Representative Rescue/Intervention	Key References
Nuclear mechanics/structural destabilization	Lmna loss-of-function and haploinsufficiency models (Lmna^−/−^, Lmna^+/−^, LmnaΔ8-11), E82K, H222P, N195K, R225X	DCM, fibrosis, conduction defects, abnormal nuclear morphology	Nuclear lamina weakening impairs nucleo–cytoskeletal coupling, desmin/connexin organization, and force transmission under contractile stress	Cardiomyocyte lamin A re-expression; exercise benefit reported in selected models	[44,45,46,48,129]
Chromatin dysregulation/transcriptional reprogramming	T10I, R541C, K117fs, Q353R, *LMNA*-haploinsufficient hiPSC-CMs, *Lmna* loss in mESCs/embryos, c.357-2A>G	Altered cardiomyocyte identity, ectopic non-cardiac gene activation, precocious differentiation, maturation defects	Disrupted LAD organization, altered compartment dynamics, and impaired lamin-dependent chromatin repression	Gata4 silencing; PDGFRB inhibition; TT-10	[40,78,130,131,132,133]
DNA damage/inflammatory stress signaling	D300N, cardiomyocyte-specific Lmna deficiency, c.357-2A>G primate model	Fibrosis, apoptosis, progressive dysfunction, arrhythmia	Nuclear damage activates DDR-TP53 and cGAS-STING inflammatory signaling	Cardiac Tp53 deletion; genetic ablation of Mb21d1/cGAS	[134,135,136]
Stress kinase and organelle homeostasis defects	H222P, R225X, R321X, R249W, E317K/N195K/Q353K	LV dysfunction, sarcomere disorganization, apoptosis, Ca^2+^ dysregulation, stress hypersensitivity, ventricular dilation	ERK1/2 and p38α MAPK hyperactivation, PERK/UPR signaling, mitochondrial/redox imbalance, broad stress-network remodeling	ERK1/2 inhibition; p38α inhibition; salubrinal; guanabenz; empagliflozin	[137,138,139,140,141,142]
Electrophysiological remodeling/arrhythmogenesis	N195K, K219T, R249Q, S143P, mechanically stretched *LMNA*-mutant hiPSC-CMs	Conduction slowing, arrhythmia, prolonged APD, EADs/DADs, abnormal Ca^2+^ handling	Altered sodium current, SCN5A/Nav1.5 repression, intercalated disk dysregulation, mechanosensitive TRPV4/TRPV2-mediated Ca^2+^ influx	Ranolazine or low-dose TTX; SCN5A overexpression; TRPV4 inhibition	[143,144,145,146,147]
Variant-specific precision rescue	K117fs, Q353R, R321X, R225X/Q354X/T518fs, K219T	Differential therapeutic responsiveness despite overlapping clinical endpoints	Dominant downstream pathway depends on variant class, dosage, splicing/NMD context, and cell state	PDGFRB inhibition; TT-10; UPR modulators; PTC124; SCN5A restoration	[131,132,140,144,148]

## Data Availability

No new data were created or analyzed in this study.
